# An Electronically Reconfigurable Highly Selective Stop-Band Ultra-Wideband Antenna Applying Electromagnetic Bandgaps and Positive-Intrinsic-Negative Diodes

**DOI:** 10.3390/mi15050638

**Published:** 2024-05-09

**Authors:** Anees Abbas, Niamat Hussain, Md. Abu Sufian, Wahaj Abbas Awan, Jaemin Lee, Nam Kim

**Affiliations:** 1 Department of Information and Communication, Chungbuk National University, Cheongju 28644, Republic of Korea; anees@chungbuk.ac.kr (A.A.); sufian@chungbuk.ac.kr (M.A.S.); wahajabbasawan@chungbuk.ac.kr (W.A.A.); biuy0882@gmail.com (J.L.); 2 Department of Intelligent Mechatronics Engineering, Sejong University, Seoul 05006, Republic of Korea; niamathussain@sejong.ac.kr

**Keywords:** antenna, high selective notch band, PIN diodes, frequency reconfigurable antenna, ultra-wideband antenna

## Abstract

In this article, an ultra-wideband (UWB) antenna featuring two reconfigurable quasi-perfect stop bands at WLAN (5.25–5.75 GHz) and lower 5G (3.4–3.8 GHz) utilizing electromagnetic bandgaps (EBGs) and positive-intrinsic-negative (P-I-N) diodes is proposed. A pair of EBG structures are applied to generate sharp notch bands in the targeted frequency spectrum. Each EBG creates a traditional notch, while two regular notches are combined to make a quasi-perfect, sharp, notch band. Four P-I-N diodes are engraved into the EBG structures to enable notch band reconfigurability. By switching the operational condition of the four diodes, the UWB antenna can dynamically adjust its notching characteristics to enhance its adaptability to various communication standards and applications. The antenna can be reconfigured as a UWB (3–11.6 GHz) without any notch band, a UWB with a single sharp notch (either at WLAN or 5G), or a UWB with two quasi-perfect notch bands. Moreover, the antenna’s notch bands can also be switched from a traditional notch to a quasi-perfect notch and vice versa. To confirm the validity of the simulated outcomes, the proposed reconfigurable UWB antenna is fabricated and measured. The experimental findings are aligned closely with simulation results, and the antenna offers notch band reconfigurability. The antenna shows a consistently favorable radiation pattern and gain. The dimension of the presented antenna is 20 × 27 × 1.52 mm^3^ (0.45 λc × 0.33 λc × 0.025 λc, where λc is the wavelength in free space).

## 1. Introduction

Ultra-wideband (UWB) antennas are designed to work over a broad spectrum of 3.1–10.6 GHz. These antennas are characterized by their wide bandwidth, which can be as much as several gigahertz. The Federal Communications Commission (FCC) has approved the use of this bandwidth (3.1–10.6 GHz) to UWB systems [1], and this spectrum continuously attracts different industries and never loses interest even when the technology evolves to the next level. The UWB system has a low power spectral density of 41.3 dBm/MHz [2], an excellent multipath channel performance, higher data rates, and an extremely large impedance bandwidth as compared to other conventional narrowband systems. The microstrip patch antennas for UWB systems are distinguished by their small footprint, affordability, featherweight, and seamless integration with other systems [3,4,5]. Most recently, this technology has been used for data collection in target sensing, exact location, and tracking, and is also employed in the latest smartphones. Before the FCC allocated the UWB band, a few narrow bands were already authorized to different technologies, such as wireless local area networks (WLANs) (5.25–5.75 GHz), and the satellite information downlinks in the 7.15–7.75 GHz band and 5G sub-6 GHz band released in July 2016 also exist within the UWB spectrum. Seeing this as an issue, it needs to be fixed to avoid interference and to undo any kind of interference caused by the previously mentioned frequency ranges. The first breakthrough was to add filters to the antenna to remove the interference. But adding filters makes the antenna size large, complex, and costly. Therefore, researchers have offered various methods that have been employed, including carving slots into the radiating element, incorporating electromagnetic bandgap (EBG) structures, and utilizing various split-ring resonators (SRR) for energy absorption [6,7,8,9,10]. By applying these techniques, single- and multi-notch-band antenna designs have been proposed in academia [11,12,13,14,15]. Although multi-band notch antennas are available, their usage is limited due to the fixed notch bands. This can be overcome by developing reconfigurable antennas. This functionality enables multi-band wireless systems, spectrum use optimization, and improved connection in a variety of communication situations.

Most recently, multi-band antennas with reconfigurable capabilities are being used in advanced microwave components as a result of the rapid expansion of wireless communication systems [16,17,18,19,20]. Switching to the required band helps in the use of a single antenna for multiple technologies, which minimizes the cost of the system and also ensures compactness. This also enables the use of multi-band notched antennas to the full extent. Because of the principles of spectrum access and cognitive radio [21,22], reconfigurable and adjustable devices offer the crucial hardware necessary for effective spectrum management and utilization. In the literature, researchers have proposed different reconfigurable antennae for different purposes such as pattern reconfigurability, polarization reconfigurability, and frequency reconfigurability [23,24,25,26,27,28,29,30]. The reconfigurability can be achieved by using diodes, micro-electro-mechanical system (MEMS) switches, and different RF switches [31,32].

The work by the authors in [12] examined a single rectangular notch band with MIMO characteristics to enhance the channel capacity of the antenna, while the reported work in [31] presents the reconfigurability of the conventional notch bands. In contrast, the present work provides a dual rectangularly notched-band reconfigurable antenna which can also be converted from conventional to rectangular notch bands depending on the needs of the system.

In [33], for smart devices, a printed ultra-wideband antenna with on-demand notching reconfiguration features has been proposed, and the reconfigurability is acquired by incorporating P-I-N diodes. To deliver reconfiguration in [34], the reconfigurable slot structures are added to the ground plane. In [35], a circularly polarized reconfigurable antenna is introduced, featuring a switchable integrated feed line. The frequency reconfiguration is achieved through the utilization of three P-I-N diodes. By using varactors in the S-SRR loaded feed line, a reconfigurable antenna is proposed in [36] by Ali Karimi et al. In [37], the notch bands are achieved by different English-alphabet-shaped open-ended stubs, while the reconfigurable characteristic is acquired by varactor diodes without any alteration in the radiation patch. The work presented in [38] describes a UWB antenna in which the rejection bands are acquired by adding a slotted split-ring resonator and the reconfigurable characteristic is achieved by adding P-I-N diodes to the SRRs.

Despite the persistent challenge of integrating multiple notches and multifunctionality into a compact antenna, this study focuses on designing a UWB antenna capable of two quasi-perfect notched bands with reconfiguration capabilities [39]. To mitigate interference between the lower 5G band and the WLAN band, the design incorporates four EBGs. The resulting reconfigurable UWB antenna achieves a −10 dB impedance spectrum range spanning from 3.1 GHz to 11.8 GHz. The authors believe that the reconfigurability of the antenna keeping the quasi-perfect stop-band characteristic is challenging and claim to achieve this for the first time. Notably, the fabricated antenna’s performance aligns remarkably well with the simulations conducted using Computer Simulation Technology (CST) 2022.

## 2. Antenna Design Procedure

### 2.1. Design of UWB Antenna with Quasi-Perfect Stop-Band Bands at 3.4–3.8 GHz and WLAN Band

**Figure 1:**
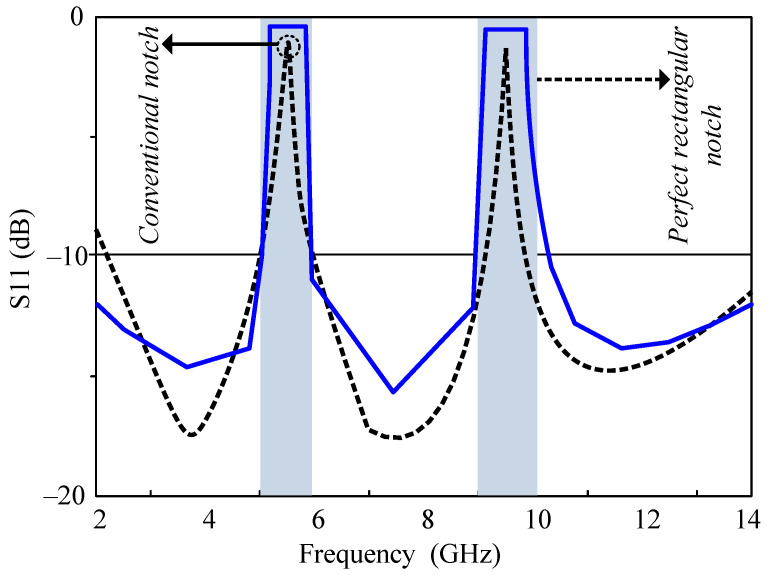
Representation of regular notch bands and quasi-perfect notch bands in a UWB antenna.

Implementing UWB antennas presents a problem when interference from coexisting technologies, such as WLAN, WiMAX, and satellite downlink–uplink frequencies, utilized by technologies such as UWB, should be prevented. To address this issue, band-notched antennas with various methods used to generate notched characteristics have been proposed by researchers. Most of the antennas have conventional notch bands; our design’s main purpose is to design an antenna with a quasi-perfect notch band to avoid interference to the maximum extent. For a better understanding of the difference between a conventional stop band and a quasi-perfect notch band, a schematic diagram is depicted in Figure 1.

Evident from the illustration is that a quasi-perfect notch is an almost perfect notch that can effectively eliminate interference across the entire range equally in entire notch bands. In this design, the stop band within the UWB antenna is achieved by EBG structures.

EBG structures belong to the category of metamaterials, serving as a valuable tool for enhancing antenna performance. These structures consist of a regular array of metal elements, such as wires or patches, which are placed on a backside insulating substrate opposite to the radiator. The design of the EBG structure can be adjusted to create a bandgap, which is a range of frequencies where the structure has an exceedingly high electrical impedance. By placing the EBG structure on the backside of an antenna, it is possible to create a stop band for certain unwanted frequencies, which can improve the overall efficiency of the antenna. For example, EBG structures have been used to reduce the level of surface electromagnetic waves and improve the performance of the antenna. The space between the radiating patch and the EBG surface is 1.52 mm, which is the height of the substrate. Then, both are connected via a shorting pin, and the substrate has a relative permittivity of 2.2.

The EBG structures in this configuration comprise four rectangular conductors which are connected to the radiating patch by the shorting of a radius ‘*p*’. Typically, these E bandgaps are crafted from small metal patches placed on dielectric substrates.

**Figure 2:**
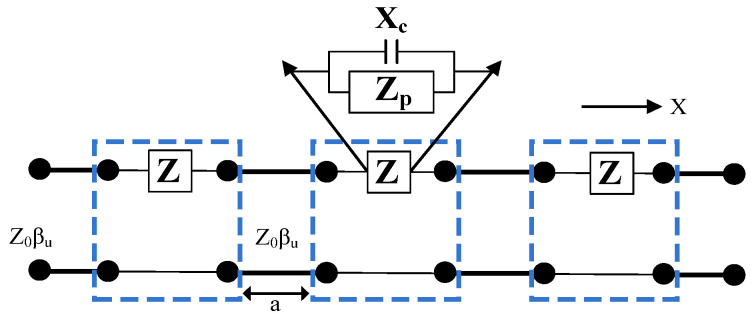
Equivalent circuit of EBG structure.

The equivalent transmission line model of the EBG structures is shown in Figure 2 [40]. The unit element of the EBG consists of a rectangular patch and a shorting pin connected with the ground. The spacing between the EBG elements results in an additional capacitance, whereas the shorting pin provides inductance. The impedance of each EBG element is denoted by Ze while the capacitive load (Xn) introduced due to the presence of neighboring elements results in an additional capacitance which can be calculated by Equation (1) [40].

(1) $$C=\left(\frac{A\varepsilon_{0}(\varepsilon_{r1}{+\varepsilon }_{r2})}{\pi }\right)cosh^{-1}\left(\frac{t}{d}\right)$$

Here, *A* is the length of the EBG, and *d* represents the distance between two consecutive EBGs, while *t* = *A* + *d*.

Moreover, the inductance generated by the shorting pin can be calculated by Equation (2).

(2) $$L=2\times 10^{-7}S_{h}\;\left[ln\left(\frac{4S_{h}\;}{g}\right)+0.5\left(\frac{g}{S_{h}\;}\right)-0.75\right]$$

Here, $S_{h}$ is the height of the substrate while g is the diameter of the via.

Equation (3) is applied to calculate the impedance ($z_{p}$) of each EBG element.

(3) $$z_{p}=z_{0}\left(\frac{z_{l}+{jz}_{0}tan(\beta_{u}l)}{z_{0}+{jz}_{1}tan(\beta_{u}l)}\right)$$

Here, *Z_l_* is the loading impedance of length *l* and *Z*_0_ stands for the characteristic impedance. By solving Equations (1)–(3), we can calculate the value of *Z* by the following relationship:

(4) $$z=\left(\frac{z_{p}x_{c}}{z_{p}+x_{c}}\right)$$

**Figure 3:**
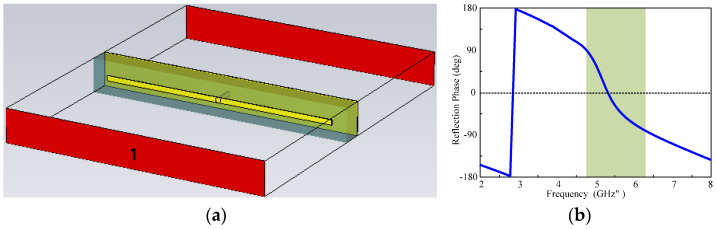
(**a**) The unit cell of EBG designed for the WLAN band and (**b**) the reflection phase of the EBG at the WLAN band.

Additionally, EBG structures can be used to create a more directive radiation pattern and to increase the isolation between antennas in a multi-antenna design. The EBG structures are used as energy absorbers at certain frequencies. The simulated results of the unit cell EBG designed for the WLAN band are shown in Figure 3. From the figure, it can be seen that the phase shift at 5.3 GHz shows that the proposed EBG structure manipulates the electromagnetic waves to create a bandgap at the targeted frequency spectrum. The proposed rectangular-shaped EBG structure shows a reflection phase 0° for a normal incidence at 5.3 GHz.

**Figure 4:**
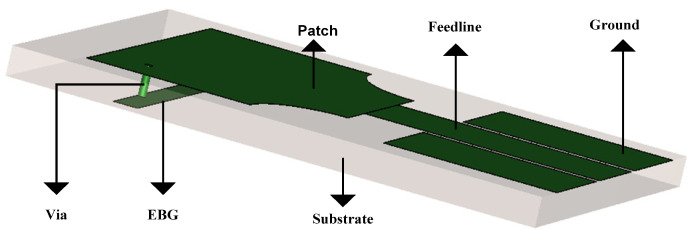
The schematic diagram shows the EBG structure connected with the patch.

In this research, four different EBG structures have been used to achieve sharp rejected bands at WLAN and lower 5G bands. The length and the width of the EBG structures, including the position on the radiation patch, help to obtain the required stop bands. The position of the shorting P-I-N on the EGB structure helps to find the notch at lower band frequencies. The EBG structure integrated with the proposed antenna is shown in Figure 4.

**Figure 5:**
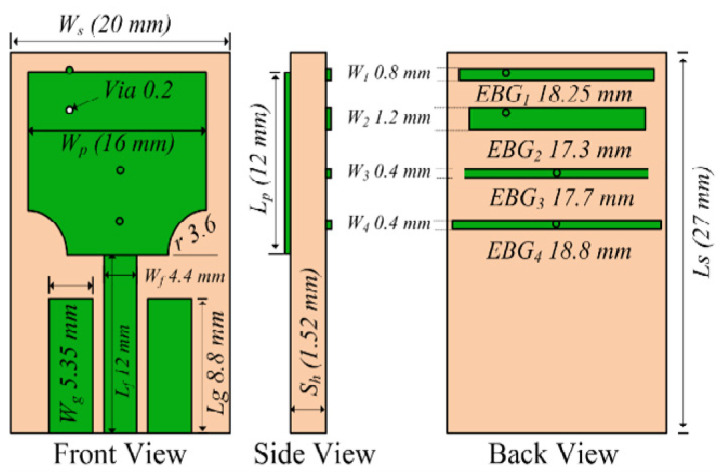
The configuration of sharp dual notch UWB antenna.

All four EBGs are the same with a difference in length and width. The radius of the shorting pins is also uniform. The EBG structures are optimized for different frequencies, for instance, width *w*, length *l*, and the position on the radiation patch *p* are optimized to achieve the stop bands. The schematic representation of the dual sharp attenuated band antenna’s design configuration can be seen in Figure 5. The phase shift shows that the proposed EBG structure manipulates the electromagnetic waves to create a bandgap. The conventional patch antennas offer a limited bandwidth of around 1–6 GHz. These antennas are useful because of their flat shape and minimum weight compared to other types of antennas. The drawback of these antennas is that they radiate at limited frequencies. Researchers have found different techniques to expand the operating frequency range of patch antennas such as etching slots, adding vias, partial grounding, and many more. Therefore, in this design, the lower corners have been cut to acquire the entire UWB bandwidth. The operational bandwidth achieved after cutting the lower ends of a radiating patch is 3.1–11.8 GHz. The antenna is printed on FR-4 (Lossy) (ɛ_r_ = 2.2, tanδ = 0.0009, h = 60 mils). The radiating patch is a square shape with the lower corners truncated. Partial grounding is employed to guarantee the antenna’s broad frequency range. The notch bands are achieved by two pairs of electromagnetic bandgap structures (EBG).

**Figure 6:**
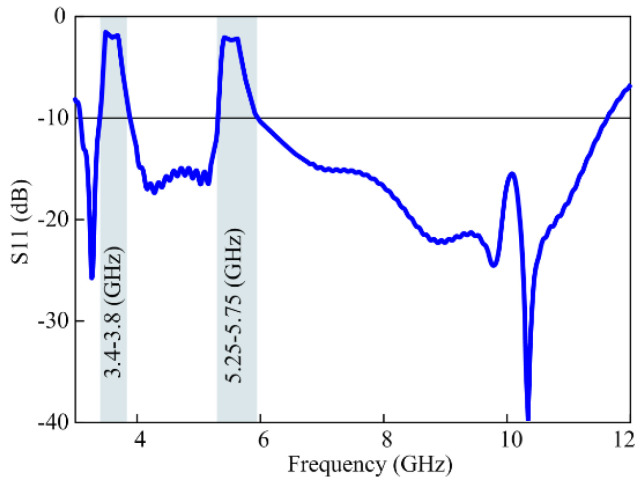
The |S_11_| results of the sharp dual quasi-perfect notch-band antenna.

A single electromagnetic bandgap structure is applied to accomplish a conventional notch of 5.4 GHz, and then another EBG is added to achieve another conventional notch near 5.4 GHz. The two conventional notched bands are then optimized to make a single quasi-perfect stop band to avoid maximum interference. Furthermore, after employing two extra bandgap structures, another sharp notch at the 5.15–5.75 GHz band is realized. The effective impedance band of the UWB antenna with both quasi-perfect notch bands is shown in Figure 6. The length and the width of the EBG structure have been optimized to acquire the desired notch bands.

### 2.2. P-I-N Diodes

An antenna that can dynamically, controllably, and irreversibly change its frequency and radiation characteristics is said to be reconfigurable. Reconfigurable antennas employ internal methods, such as varactors, RF switches, mechanical actuators, or configurable materials, to allow deliberate manipulation of current distribution across the antenna’s surface. This results in reversible adjustments to its properties, enabling dynamic responsiveness. Unlike smart antennas, which rely on an external beamforming network, reconfigurable antennas integrate the reconfiguration mechanism directly into the antenna structure. This integration allows reconfigurable antennas to adapt and optimize their performance in response to changing environmental conditions or evolving operational requirements. Antennas with frequency reconfigurability can dynamically change their operating frequency. Because the numerous antenna requirements can be substituted by a single antenna, they are especially helpful in scenarios where multiple communications systems converge. RF switches are typically used to change the antenna’s size physically or electrically to reconfigure the frequency. Several types of electronic devices can be used to implement a reconfigurable antenna. As a varactor diode, these semiconductor devices can be used to change the capacitance of an antenna, which can be used to modify the impedance spectrum range of the antenna. This can be achieved by applying a voltage to the diode; consequently, it alters the depletion region’s width, affecting the capacitance.

**Figure 7:**
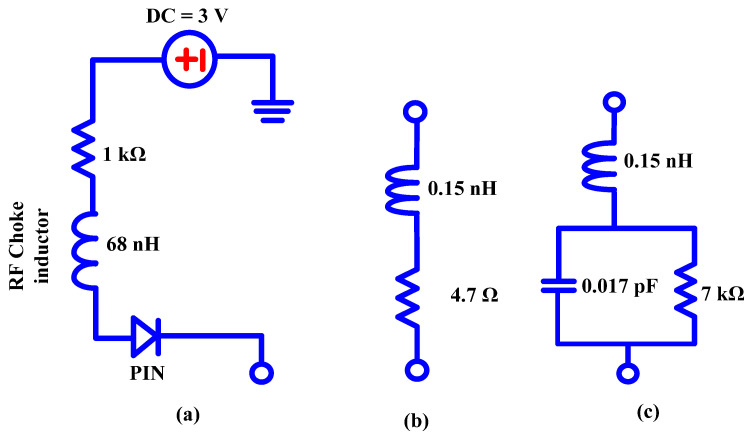
Equivalent circuit of a P-I-N diode: (**a**) the DC circuit diagram of P-I-N diode, (**b**) forward-biased, (**c**) reverse-biased.

P-I-N diodes are three-layer semiconductor devices used in multiple applications, including notch-band implementations in antenna design systems. The layers of the diode, the P-region, I-region (intrinsic), and N-region, give the acronym “PIN” its meaning. These diodes have special qualities that make them appropriate for notch-band applications. The ability of PIN diodes to reverse bias is one of their key characteristics. The PIN diode enters a low-impedance state when a forward bias is applied across it, allowing current to pass through. Applying a reverse bias, on the other hand, raises the diode’s resistance and limits the current flow. This property renders it most suitable for applications involving reconfigurable antennas. A DC circuit diagram of the P-I-N diode is presented in Figure 7 [41].

The diodes have two biasing arrangements: forward bias and reverse bias. Forward biasing is the process of connecting a diode so that the voltage across it enables current to flow from the cathode (negative terminal) to the anode (positive terminal) in the forward direction. Applying a higher voltage to the cathode and a positive voltage to the anode often results in this. For typical diode functioning, such as in rectifiers, a forward bias is used. Reverse biasing is the process of connecting a diode to a voltage that stops current from flowing from the cathode to the anode in the opposite direction. By applying a larger voltage to the cathode and a lower voltage to the anode, this is accomplished. The breakdown voltage and leakage current characteristics of the diode are frequently investigated using reverse bias. The precise application and desired behavior of the diode inside a circuit determine the biasing arrangement that should be used. Diode behavior can be regulated using a variety of setups, such as voltage regulation or rectification.

### 2.3. Design of Reconfigurable UWB Antenna

**Figure 8:**
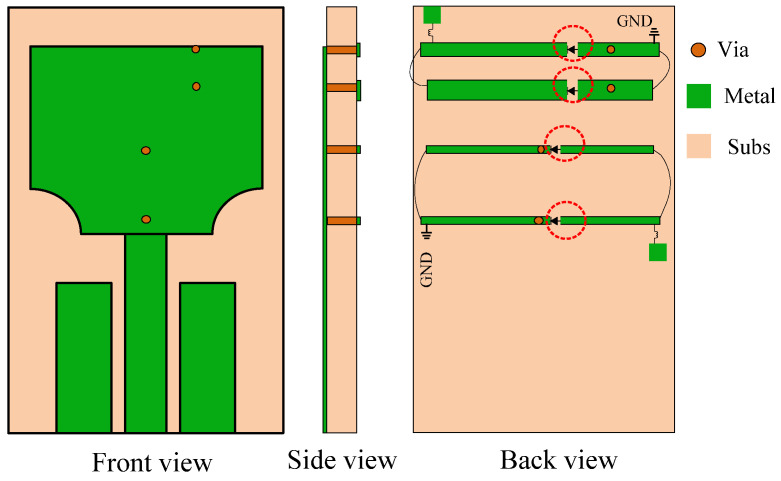
The geometry of the proposed dual-notch-band UWB antenna.

In this study, we have employed P-I-N diodes to reconfigure the UWB antenna into four distinct configurations. These configurations are attained by toggling the biasing of the four P-I-N diodes, allowing the antenna to function as a UWB antenna, a UWB antenna offering a single rejected band targeting either the WLAN band or the 5G Sub-6 GHz band, or a UWB antenna with notches in both frequency bands. Subsequent sections will delve into the details of these four different operational states. Figure 8 illustrates the geometry of the designed antenna, comprising a radiating patch, a partial ground plane, four EBG structures, and four P-I-N diodes. Each of the P-I-N diodes integrated into the EBG structure is denoted as diode 1, diode 2, diode 3, or diode 4. The two EBGs seen on the top are utilized to create a notch in the 5G Sub-6 GHz band, while the third and fourth EBGs are employed to block the WLAN frequency band.

### 2.4. Reconfigurable Characteristics of Notched-Band Antenna

#### 2.4.1. Case 1: UWB Antenna without Any Rejection Band

**Figure 9:**
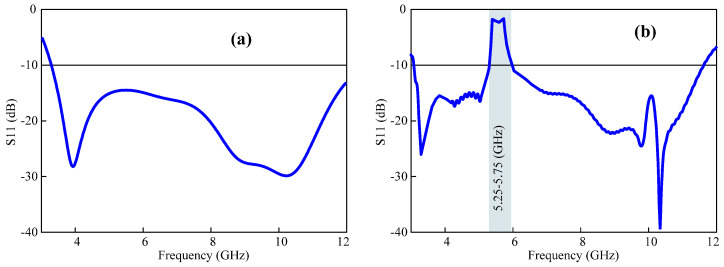
Reflection coefficient of the antenna (**a**) without any stop band and (**b**) coefficient with WLAN-restricted frequency range.

A UWB antenna is engineered with dual stop bands, enabling its operation across a broad frequency spectrum, typically spanning from 3.1 GHz to 11.8 GHz, but with the ability to exclude certain frequencies that are used by other communication technologies such as WLAN (5.25–5.75 GHz) band and 5G sub-6 (3.4–3.8 GHz). This is accomplished by incorporating four diodes into the EBG structure of the antenna. The reflection coefficient for case 1 is shown in Figure 9a. The integrated P-I-N diodes on EBGs are reverse-biased, and the antenna works in the whole designated UWB bandwidth without filtering any interference. The biasing states of the P-I-N diodes are shown in Table 1, given below.

#### 2.4.2. Case 2: Reconfiguration of UWB Antennas with WLAN Stop Bands

The configuration of the P-I-N diodes for case 2 is shown in Table 1. By turning off the biasing state of two diodes engraved on EBG1 and EBG2, the antenna will be applicable to all of the operating bandwidths except the WLAN band. The reflection factor of a single quasi-perfect stop band, which is case 2, is illustrated in Figure 9b.

#### 2.4.3. Case 3: Reconfiguration of UWB Antennas with 5G Sub-6 GHz Stop Band

**Figure 10:**
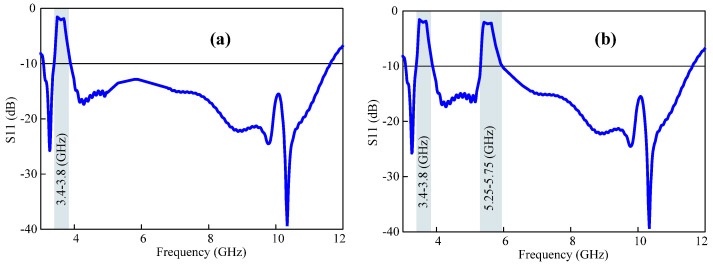
The reflection coefficient of the antenna (**a**) with a lower 5G rejection band. (**b**) Notch bands at lower 5G GHz and WLAN bands.

In this case, the antenna can be reconfigured to a UWB antenna with a lower 5G stop spectrum. The P-I-N diode biasing states are depicted in Table 1. The P-I-N diodes installed to the EGB structure which are used to stop the interference of the WLAN band have been turned off. This enables the utilization of the antenna with a quasi-perfect single rejected band at the 5G Sub-6 GHz frequency spectrum. Figure 10a illustrates the reflection coefficient for case 3.

#### 2.4.4. Case 4: Reconfigurable Dual Quasi-Perfect Stop-Band UWB Antenna

The two extremely selective stop bands are made possible by the P-I-N diode’s biasing configuration. To reject interference with the quasi-perfect notch in both the WLAN and lower 5G bands, all P-I-N diodes have been forward-biased to activate the EBGs. In Table 1, the possible reconfigurable states are given. The antenna featuring dual sharp notch bands exhibits its |S11| characteristic in Figure 10b.

#### 2.4.5. Controllability of Stop-Band Bandwidth

The incorporation of P-I-N diodes has proven particularly valuable in antenna design. This has provided effective controllability of the antenna. The control of stop bandwidths can also be achieved by manipulating the conditions of all diodes. A pair of EBG structures have been employed to achieve a sharp notch band, which is the addition of two different conventional stop bands. Thus, these stop bands can be adjusted by altering the biasing state. This can be accomplished by changing the biasing states of the P-I-N diodes employed to create sharp notch bands. We have added four P-I-N diodes, which provide 12 flexible states to the proposed antenna. The four main states were discussed earlier. In the later part, we will discuss the remaining states that are applicable to controlling the notch-band bandwidth. It is worth noting that all 12 states could be used when needed.

**Figure 11:**
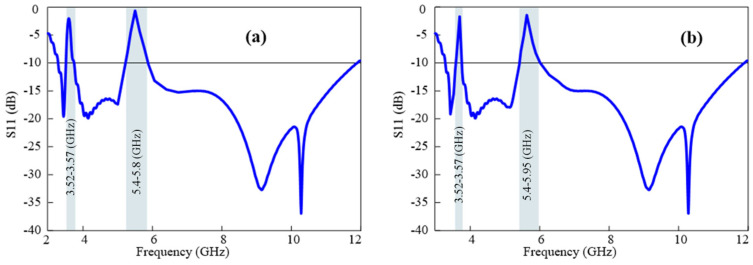
The reflection antenna when (**a**) diodes 1 and 3 are OFF and (**b**) diodes 2 and 4 are OFF.

When the biasing states of diodes 1 and 3 are set to OFF, both notch bands will function as conventional with the given notching bandwidths of the WLAN band (5.4–5.95 GHz) and the 5G sub-6 GHz (3.52–3.7 GHz). The |S11| response of the antenna is shown in Figure 11a. The notch bands are not as selective compared with the two EBG structures. When the biasing states of diodes 2 and 4 are set to OFF, the antennas will again be functional due to the dual conventional notch bands with the given notching bandwidths for the WLAN band (5–5.5 GHz) and the 5G sub-6 GHz range (3.39–3.6 GHz). The |S_11_| characteristic of the conventional stop-band antenna is presented in Figure 11b.

**Figure 12:**
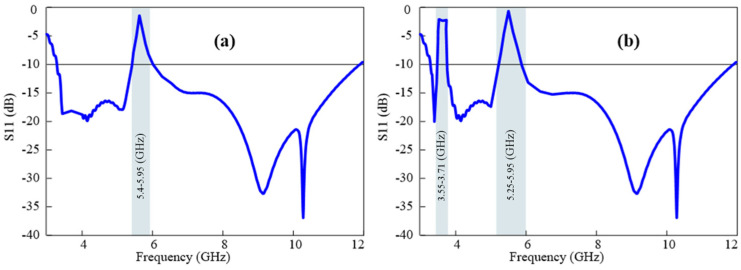
Notch bandwidth flexibility: (**a**) single conventional notch band at WLAN band by setting three diodes to OFF and diode 4 to ON, and (**b**) a sharp notch band and a conventional notch band when three diodes are in ON state.

In other scenarios, the suggested design can function with a conventional rejected-band UWB antenna by switching forward biasing to reversed biasing for three diodes for a single traditional notch-band UWB antenna. Figure 12a illustrates the conventional notch band at the WLAN frequency, achieved by altering the biasing states of the diodes. By toggling the states of the four P-I-N diodes, this proposed antenna can be reconfigured into a total of 12 different operational states. Further details about the remaining states can be found in Table 2 and Table 3.

If only one P-I-N diode is forward-biased and the remaining three are reversed-biased, there could be four reconfigurations where the antenna can serve as a single conventional notch at any band with a smaller attenuation region, as shown in Figure 12b. The diode setup configuration is presented in Table 2.

In Table 1 and the above sections, we discuss the configuration of the antenna when the two diode biasing states are varied. The antenna can also be employed in a configuration featuring a nearly perfect stop band alongside a conventional notch band. In this configuration, three diode states are active while a single diode remains in the inactive state. The configuration of the diodes is shown in Table 3, and for a better picture, in Figure 10b a quasi-perfect stop band at the lower-frequency 5G band and a conventional notch band at the WLAN band is depicted.

## 3. Results and Discussion

**Figure 13:**
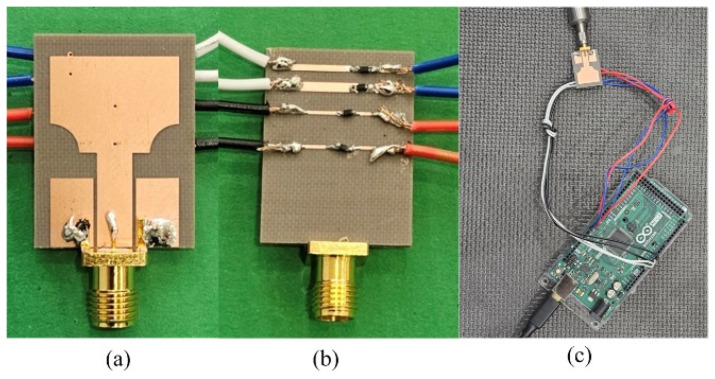
The fabricated reconfigurable UWB antenna: (**a**) frontside, (**b**) backside, (**c**) antenna with controller circuitry.

The design of the reconfigurable UWB antenna was carried out using the electromagnetic simulation software Computer Simulation Technology 2022 (CST) and was subsequently implemented on a cost-effective Taconic TLY-5 lossy substrate for result verification. To obtain accurate measured results, the antenna was fed with an SMA connector in the simulations. Figure 13 illustrates the physical embodiment of the fabricated reconfigurable UWB antenna.

**Figure 14:**
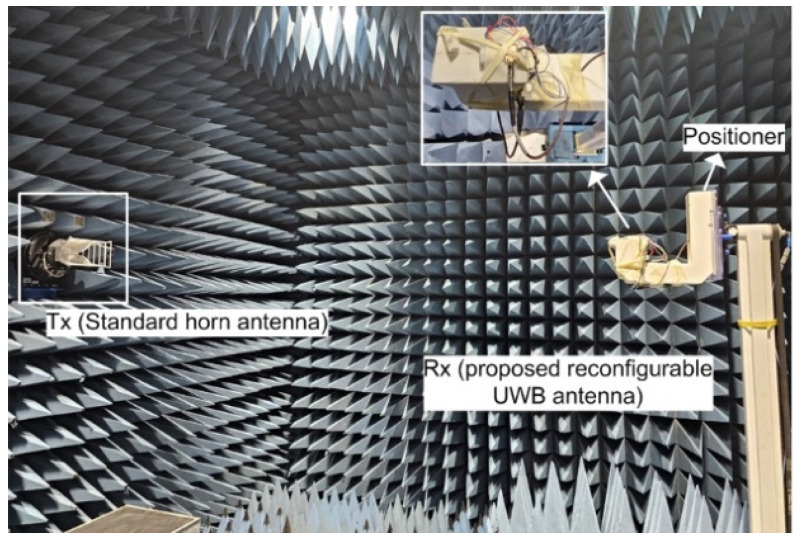
Radiation measurement setup of fabricated UWB antenna.

The dimensions of the antenna are 20 × 26.7 × 1.52 mm^3^. The antenna’s radiation patterns and gain were evaluated in an anechoic far-field chamber, as depicted in Figure 14.

**Figure 15:**
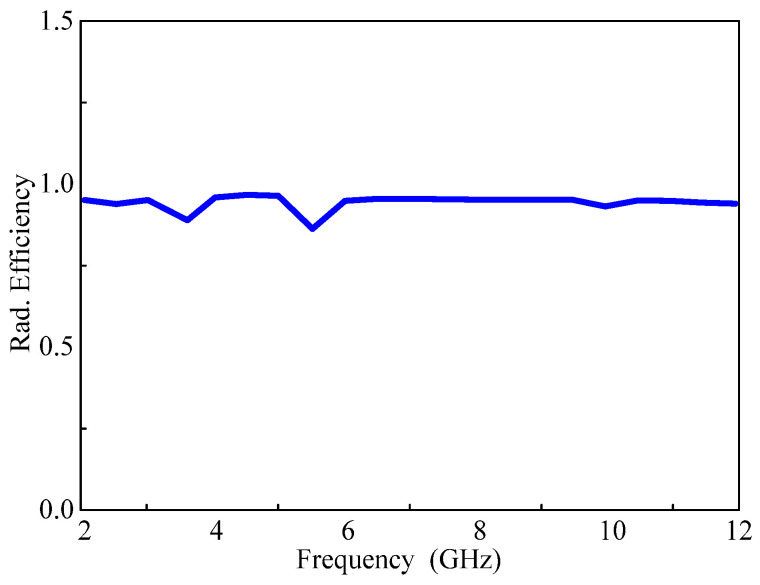
The overall efficiency of the proposed reconfigurable antenna.

**Figure 16:**
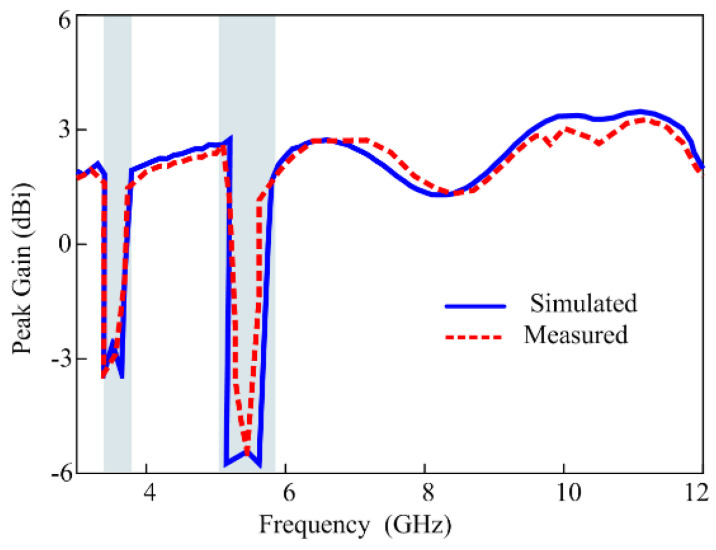
Simulated versus measured gains of the proposed antenna.

The overall radiation efficiency averages at 98 percent, except within the stop band where the radiation efficiency experiences a significant degradation, as illustrated in Figure 15. For a comprehensive view of the antenna’s performance, both the simulated and measured gains are displayed in Figure 16. As illustrated in the figure, the simulation gain results show a strong correlation with the measured gain results. The gains at both stop bands drop sharply when all diodes are forward-biased. The antenna achieves its highest gain of 3.5 dBi at 11 GHz, while the entire passband has more than 1.5 dB gain except for the notch bands where the minimum gains are −1.1 dB and −0.7 dB at the WLAN and 5G sub-6 GHz bands, respectively.

**Figure 17:**
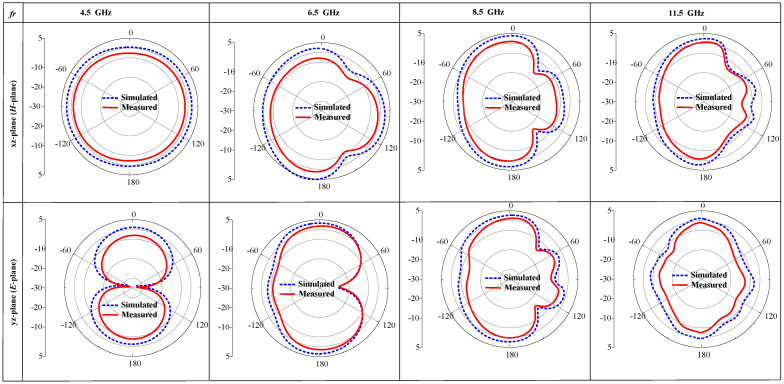
Analyzed simulated radiation behavior in the E- and-H-planes at frequencies 4.5, 6.5, 8.5, and 11.5 GHz.

Subsequently, in Figure 17, we present both the simulation and practically examined radiation patterns in both planes (E and H) at frequencies 4.5, 6.5, 8.5, and 11.5 GHz. In the xz-plane, the antenna shows a circular radiation pattern, while in the xy-plane, it resembles a dumbbell shape. The radiation pattern is distorted at higher frequencies since the equivalent radiating area increases at high frequencies, and the wavelength of the electromagnetic waves becomes smaller and more sensitive to the surrounding environment. Also, the cables in diode biasing, the SMA connection, and the cable connected to the antenna ground plane are among the variables that cause differences between the findings of the simulations and the actual tests. Remarkably, the reconfigurable antenna shows similar radiation patterns, as the utilization of P-I-N diodes does not significantly affect the radiator.

**Figure 18:**
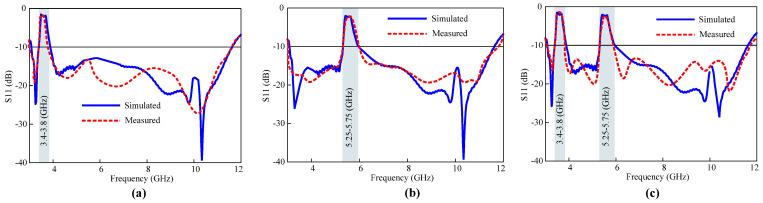
Simulated and observed results with (**a**) a sharp notch at 5G sub-6 GHz band, (**b**) a single sharp notch at WLAN band, and (**c**) dual rejected bands.

Additionally, three different cases in terms of the reflection coefficient of the fabricated reconfigurable UWB antenna were measured to analyze the agreement of the results with the simulated results. Figure 18a shows the results when only the 5G sub-6 GHz band is notched while the WLAN band’s interference is neglected. This can be achieved by making diodes 1 and diodes 2 forward-biased. In the second case, the states of diodes 1 and 2 are reverse-biased, which reconfigures the antenna to a single sharp notch at the WLAN band, as shown in Figure 18b. In the third case, all P-I-N diodes are forward-biased. As depicted in Figure 18c, the antenna serves as a dual quasi-perfect notch band. The measured antenna’s performance closely aligns with the simulation results, as evident from the figures.

**Figure 19:**
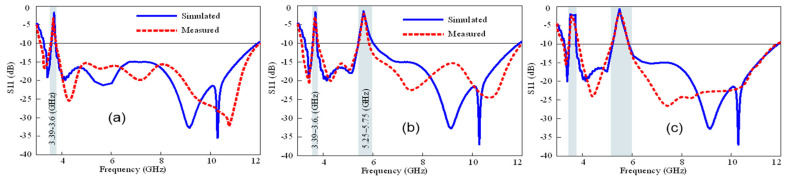
Simulated and observed results of an antenna: (**a**) single conventional notch band at 5G sub-6 GHz when P-I-N diode 1 is forward-biased, (**b**) dual conventional notch bands at WLAN band and 5G sub-6 GHz when P-I-N diodes 1 and 3 are forward-biased, and (**c**) a sharp notch band and a conventional notch band when all three diodes are forward-biased.

Finally, the reconfiguration of the stop-band bandwidths was experimentally verified. The antenna in such cases functions as a conventional rejected-band UWB antenna with varying notch bandwidths depending on the biasing states of the P-I-N diodes. Figure 19a illustrates that when a single P-I-N diode is forward-biased, the antenna rejects the smaller portion of the frequency spectrum, forming sharp notch bands. By altering the biasing states of the diodes, the presented antenna has the potential to show four different single conventional notch bands. Figure 19b highlights the antenna’s ability to be reconfigured for two conventional notch bands at the WLAN and sub-6 5G GHz bands. Furthermore, Figure 19c demonstrates the antenna’s capability to operate with a single sharp band and conventional notch bands simultaneously.

Compared to the other UWB antennas with reconfigurable characteristics, the proposed antenna stands out with its unique quasi-perfect notch-band characteristics. The capability to reconfigure both the frequency and bandwidth of the notch bands makes it highly versatile and the high selectivity of two notch bands allows for precise control over the antenna’s behavior. While the other reconfigurable UWB antenna also offered similar frequency reconfiguration capabilities, the addition of two quasi-perfect notch bands is a distinguishing factor that sets the proposed reconfigurable UWB antenna apart.

A comparative analysis has been conducted between previously published antennas and the presented antenna given in Table 4 in terms of antenna dimensions (mm^2^), bandwidth, notch bandwidth, notch selectivity (rejects the interference from the notch band almost equally), notch controllability, and notch band minimum gain (dB).

## 4. Conclusions

In this article, a UWB antenna with reconfigurable dual-sharp notched-band characteristics has been suggested. The notching characteristics are achieved by applying EBG structures, and the sharp notch is realized by tuning the parameters of a pair of EBG structures. The reconfigurability of the proposed UWB antenna is attained by varying the biasing states of the four P-I-N diodes that are integrated into the EBGs. By managing the biasing of these diodes, the antenna’s behavior and performance can be modified and adapted to different operating conditions and frequency requirements The proposed antenna is suitable for a variety of applications for the entire UWB spectrum (3.1–11.8 GHz); for a UWB with dual sharp notch bands (WLAN band and 5G sub-6 GHz band); and for a UWB with single quasi-perfect notch bands, either of the wireless local area network or the 5G sub-6 GHz bands. The bandwidth of the rejected bands can also be managed by altering the biasing of diodes. The proposed quasi-stop-band reconfigurable antenna can be a potential candidate for technologies using the UWB spectrum with its compact size of 26 × 20 × 1.52 mm^3^.

## Figures and Tables

**Table 1:** The P-I-N diode state for case 1.

States	D1	D2	D3	D4	Notch Bands	Characteristics
I	OFF	OFF	OFF	OFF	None	Entire UWB band
II	ON	ON	ON	ON	Dual notch band	Dual-notch-band UWB antenna
III	ON	ON	OFF	OFF	Single	Single-notch-band UWB antenna
IV	OFF	OFF	ON	ON	Single	Single-notch-band UWB antenna

**Table 2:** The P-I-N diode state for single convention notch band.

States	D1	D2	D3	D4	Notch Bandwidth (GHz)	Characteristics
I	OFF	OFF	OFF	ON	5.4–5.95	Conventional WLAN notched band
II	OFF	OFF	ON	OFF	5–5.5	Conventional WLAN notched band
III	OFF	ON	OFF	OFF	3.55–3.71	Conventional 5G notched band
IV	ON	OFF	OFF	OFF	3.39–3.6	Conventional 5G notched band

**Table 3:** The P-I-N diode states for a sharp notch band and a conventional notch band.

States	D1	D2	D3	D4	Notch Bandwidth (GHz)	Characteristics
I	ON	ON	ON	OFF	3.4–3.8, 5–5.5	Sharp 5G and regular WLAN notched
II	ON	ON	OFF	ON	3.4–3.8, 5.4–5.95	Sharp 5G and regular WLAN notched
III	ON	OFF	ON	ON	3.55–3.71, 5.25–5.75	Regular 5G and sharp WLAN notched
IV	ON	OFF	OFF	OFF	3.39–3.6, 5.25–5.75	Regular 5G and sharp WLAN notched

**Table 4:** Performance comparison with previously reported work.

Ref.	Ant. Dimensions (mm^2^)	Bandwidth GHz	Notch Bandwidth GHz	Notch Selectivity	Notch Band Controllability	Notch Band Minimum Gain (dB)
[42]	24 × 24	3.1–10.6	3.1–3.65 4.9–5.56 5.9–6.4 7.3–8.5	low	no	−1 | −4 | −1 | 1
[43]	44.1 × 60	0.68–16.23	3.3–3.80 5.15–5.35	low	no	−6 | −7
[44]	49.4 × 35	5–24.5	N/A	low	no	Not provided
[45]	58 × 60	3.4–10.2	4.7–5.1 5.3–6.0	low	no	Not provided
[46]	22 × 13	2.82–13.25	3.19–4.58 5.26–6.21 7.87–8.73	low	no	−6 | −1 | −3
[47]	50 × 50	2.6–10.8	6.66, 8.34 9.85	low	no	Not provided
[48]	50 × 50	2.89–4.07 5.1–6.19	N/A	N/A	N/A	Not provided
[49]	27 × 33	5–6.1	5.6	low	low	−1.5
Prop.	26 × 20	3–11.6	3.4–3.8 5.25–5.75	high	yes	−3 | −5.8

## Data Availability

All data generated or analyzed during this study are included in this manuscript. There are no additional data or datasets beyond what is presented in the manuscript.
